# Aquaporin-11 Contributes to TGF-β1-induced Endoplasmic Reticulum Stress in Human Visceral Adipocytes: Role in Obesity-Associated Inflammation

**DOI:** 10.3390/cells9061403

**Published:** 2020-06-04

**Authors:** Gema Frühbeck, Inmaculada Balaguer, Leire Méndez-Giménez, Víctor Valentí, Sara Becerril, Victoria Catalán, Javier Gómez-Ambrosi, Camilo Silva, Javier Salvador, Giuseppe Calamita, María M. Malagón, Amaia Rodríguez

**Affiliations:** 1 Metabolic Research Laboratory, Clínica Universidad de Navarra, 31008 Pamplona, Spain; gfruhbeck@unav.es (G.F.); ibalaguer@alumni.unav.es (I.B.); lmgimenezde@gmail.com (L.M.-G.); sbecman@unav.es (S.B.); vcatalan@unav.es (V.C.); jagomez@unav.es (J.G.-A.); 2 CIBER Fisiopatología de la Obesidad y Nutrición (CIBEROBN), Instituto de Salud Carlos III, 28029 Madrid, Spain; vvalenti@unav.es (V.V.); csilvafr@unav.es (C.S.); jsalvador@unav.es (J.S.); bc1mapom@uco.es (M.M.M.); 3 Obesity and Adipobiology Group, Instituto de Investigación Sanitaria de Navarra (IdiSNA), 31008 Pamplona, Spain; 4 Department of Endocrinology & Nutrition, Clínica Universidad de Navarra, 31008 Pamplona, Spain; 5 Department of Dermatology, Gregorio Marañón General University Hospital, 31008 Madrid, Spain; 6 Department of Surgery, Clínica Universidad de Navarra, 31008 Pamplona, Spain; 7 Department of Biosciences, Biotechnologies and Biopharmaceutics, University of Bari “Aldo Moro”, 70125 Bari, Italy; giuseppe.calamita@uniba.it; 8 Department of Cell Biology, Physiology, and Immunology, IMIBIC/University of Córdoba/Reina Sofía University Hospital, 14004 Córdoba, Spain

**Keywords:** aquaporins, obesity, endoplasmic reticulum stress

## Abstract

Aquaporin-11 (AQP11) is expressed in human adipocytes, but its functional role remains unknown. Since AQP11 is an endoplasmic reticulum (ER)-resident protein that transports water, glycerol, and hydrogen peroxide (H_2_O_2_), we hypothesized that this superaquaporin is involved in ER stress induced by lipotoxicity and inflammation in human obesity. AQP11 expression was assessed in 67 paired visceral and subcutaneous adipose tissue samples obtained from patients with morbid obesity and normal-weight individuals. We found that obesity and obesity-associated type 2 diabetes increased (*p* < 0.05) AQP11 mRNA and protein in visceral adipose tissue, but not subcutaneous fat. Accordingly, *AQP11* mRNA was upregulated (*p* < 0.05) during adipocyte differentiation and lipolysis, two biological processes altered in the obese state. Subcellular fractionation and confocal microscopy studies confirmed its presence in the ER plasma membrane of visceral adipocytes. Proinflammatory factors TNF-α, and particularly TGF-β1, downregulated (*p* < 0.05) AQP11 mRNA and protein expression and reinforced its subcellular distribution surrounding lipid droplets. Importantly, the *AQP11* gene knockdown increased (*p* < 0.05) basal and TGF-β1-induced expression of the ER markers ATF4 and CHOP. Together, the downregulation of AQP11 aggravates TGF-β1-induced ER stress in visceral adipocytes. Owing to its “peroxiporin” properties, AQP11 overexpression in visceral fat might constitute a compensatory mechanism to alleviate ER stress in obesity.

## 1. Introduction

Aquaporins (AQPs) are membrane channels that facilitate the movement of water across biological membranes [1]. AQP structure is composed of six transmembrane α-helices and two reentrant loops with two asparagine–proline–alanine (NPA) signature motifs, which create the aperture of a water channel pore-forming a tridimensional “hourglass” structure in the lipid bilayer [2]. Based on their permeability, AQPs are classically subdivided into “orthodox aquaporins” (AQP0, AQP1, AQP2, AQP4, AQP5, AQP6, and AQP8), which are mainly pure water channels [3,4], and “aquaglyceroporins” (AQP3, AQP7, AQP9, and AQP10), which are permeated by water and other small solutes, such as glycerol, urea or nitric oxide [5]. A third subfamily of AQPs, unorthodox or “superaquaporins” (AQP11 and AQP12), exhibit unique Asn-Pro-Cys (NPC) motifs in AQP11 and Asn-Pro-Thr (NPT) in AQP12, and are located in the membranes of intracellular organelles [6,7,8]. This archetypical classification based on the transporting properties has been recently blurred because AQP11 is permeated by water [8], glycerol [9], and hydrogen peroxide (H_2_O_2_) [10]. Moreover, this ability to transport H_2_O_2_ is also observed in several orthodox AQPs (AQP5 and AQP8) [11,12] and aquaglyceroporins (AQP3 and AQP9) [13,14] that, together with superaquaporin AQP11 [10], are collectively termed “peroxiporins”. H_2_O_2_ acts as a signaling molecule in various cellular processes, including endoplasmic reticulum (ER) redox homeostasis and signaling, and therefore, these AQPs with peroxiporin activity are a matter of intense investigation in all human organs and tissues [10]. 

AQP11 (originally named AQPX1) is highly expressed in testis and, to a lesser extent, in the kidney, liver, brain, and adipose tissue [9,15]. AQP11 functions as an ER-resident peroxiporin, and its downregulation perturbs the flux of H_2_O_2_ through the ER, but not through the mitochondrial or plasma membranes [10]. In line with this observation, transgenic *Aqp11*-knockout mice die before weaning with progressive vacuolization and cyst formation of the proximal tubule, leading to lethal polycystic kidney disease with marked signs of oxidative stress [16,17,18]. These ER stress-related vacuoles are also observed in hepatocytes of liver-specific *Aqp11*-knockout mice [19]. Thus, AQP11 constitutes an important regulator of renal and hepatic ER redox homeostasis and signaling. 

Obesity is a condition of low-grade chronic inflammation. Failure of the unfolded protein response (UPR), an ER stress response to restore protein homeostasis, is one of the signals that perpetuate the chronic inflammation in adipose tissue and liver in obesity [20,21,22], but little is known about the mechanisms triggering obesity-induced ER stress. The ER-resident AQP11 was identified close to lipid droplets in human adipocytes [8], but its function remains undefined. Therefore, our hypothesis was that AQP11 is involved in the relationship between ER stress and chronic inflammation in the context of human obesity and obesity-associated type 2 diabetes (T2D). To gain insight into the regulation of AQP11, the role of tumor necrosis factor α (TNF-α) and transforming growth factor β1 (TGF-β1), two molecules tightly involved in obesity-associated inflammation and ER stress, was analyzed as plausible regulators of AQP11 in human omental adipocytes.

## 2. Materials and Methods

### 2.1. Patient Selection

Paired omental and subcutaneous adipose tissue samples (n = 67) were collected from patients undergoing either laparoscopic Roux-en-Y gastric bypass for morbid obesity treatment (n = 53, patients with obesity) or laparoscopic hiatal hernia repair with Nissen fundoplication (n = 14; control lean patients) at the Clínica Universidad de Navarra. Body mass index (BMI) was calculated as weight in kilograms divided by the square of height in meters, and body fat (BF) was estimated by air-displacement plethysmography (Bod-Pod^®^; Life Measurements, Concord, CA, USA). Obesity was classified according to both BMI (≥30 kg/m^2^) and %BF (BF ≥25% in males and ≥35% in females). Obese patients were sub-classified into two groups (normoglycemia (NG) or impaired glucose tolerance (IGT)/T2D) following the criteria of the Expert Committee on the Diagnosis and Classification of Diabetes [23]. It has to be stressed that the patients included in the T2D group did not have a long diabetes history (less than 2–3 years or even de novo diagnosis, as evidenced by their anamnesis and biochemical determinations). Biochemical and hormonal assays performed in the study subjects were measured as previously described [24]. All reported investigations were carried out in accordance with the principles of the Declaration of Helsinki, as revised in 2013, by the Hospital’s Ethical Committee (protocol 2017.104, approved in June 2017). Informed consent from all volunteers was obtained.

### 2.2. Adipose Tissue Handling

Paired samples of omental and subcutaneous fat samples were collected during elective surgical procedures and immediately stored at −80 °C for gene and protein expression analyses. A portion of omental and subcutaneous adipose tissue was fixed in 4% formaldehyde for histological analyses. Another portion of omental fat was used for the isolation of adipocytes and stromal vascular fraction cells (SVFC) by 2% collagenase digestion, as previously described [24]. Total RNA isolation and purification was performed using QIAzol^®^ Reagent (Qiagen, Hilden, Germany) and RNeasy Lipid Tissue Mini Kit (Qiagen) for adipose tissue and adipocytes, and TRIzol^®^ Reagent (Invitrogen, Paisley, UK) and RNeasy Mini Kit (Qiagen) for SVFC, according to the manufacturer’s instructions. Finally, 200 mg of omental and subcutaneous adipose tissue was homogenized in ice-cold lysis buffer (0.1% SDS, 1% Triton X-100, 5 mmol/L EDTA·2H_2_O, 1 mol/L Tris-HCl, 150 mmol/L NaCl, 1% sodium deoxycholate, pH 7.40) supplemented with a protease inhibitor cocktail (Complete^TM^ Mini-EDTA free, Roche, Mannheim, Germany). Lysates were centrifuged at 16,000× *g* at 4 °C for 15 min to remove nuclei and unruptured cells. Total protein concentrations were determined by the Bradford assay, using bovine serum albumin (BSA) (Sigma, St Louis, MO, USA) as standard.

### 2.3. Adipocyte Cultures

Human omental SVFC were seeded at 2 × 10^5^ cell/cm^2^ and grown in adipocyte medium (DMEM/F-12 (1:1); Invitrogen), 17.5 mmol/L glucose, 16 µmol/L biotin, 18 µmol/L panthotenate, 100 µmol/L ascorbate and antibiotic-antimycotic] supplemented with 10% newborn calf serum (NCS). After 4 days, the medium was changed to adipocyte medium supplemented with 3% NCS, 0.5 mmol/L 3-isobutyl-1-methylxanthine (IBMX), 0.1 µmol/L dexamethasone, 1 µmol/L BRL49653 and 10 µg/mL insulin. After a 3-day induction period, cells were fed every 2 days with the same medium but without IBMX and BRL49653 supplementation for the remaining 7 days of adipocyte differentiation. Differentiated adipocytes were serum-starved for 24 h and then treated with TNF-α (1, 10, and 100 ng/mL) (PeproTech EC, Inc., Rocky Hill, NJ, USA), TGF-β1 (0.1, 1, and 10 ng/mL) (Peprotech), insulin (1, 10, and 100 nmol/L) (Sigma) and isoproterenol (10 µmol/L) (Sigma) for 24 h. One sample per experiment was used to obtain control responses in the presence of the solvent.

### 2.4. Subcellular Fractionation Studies

Lipid droplets (LDs), cytosolic, and crude membrane fractions were isolated by centrifugation of protein extracts from differentiated adipocytes in sucrose density gradients according to our previously validated protocols [25,26]. Briefly, cells were rinsed with Ca^2+^- and Mg^2+^-free PBS (Invitrogen) and resuspended in 3 mL lysis buffer containing 25 mmol/L Tris-HCl, 100 mmol/L KCl, 1 mmol/L EDTA, 5 mmol/L EGTA, and 1 µg/mL anti-protease cocktail (pH 7.40). Cells were disrupted and mixed with an equal volume of lysis buffer containing 1.08 mol/L sucrose, and extracts were centrifuged at 1500× *g* for 10 min. Supernatants were transferred to a 12 mL ultracentrifuge tube and sequentially overlaid with 2 mL each of 0.27 mol/L and 0.135 mol/L sucrose buffer and, finally, free-sucrose solution containing 25 mmol/L Tris-HCl, 1 mmol/L EDTA, and 1 mmol/L EGTA (pH 7.40). After centrifugation at 130,000× *g* at 4 °C for 1 h, protein distribution was analyzed by Western-blot using 50 μg from the fractions containing LDs (fractions 1–2), cytosol (5–7) and membranes (8–9).

### 2.5. AQP11 Knockdown by siRNA Transfection

MISSION^®^ esiRNA targeting human *AQP11* mRNA (EHU037771) and MISSION^®^ siRNA Universal negative control number 1 (SIC001) were purchased from Sigma–Aldrich. MISSION^®^ esiRNA are a heterogeneous mixture of siRNAs that all target the same mRNA sequence, which conducts highly specific and effective gene silencing. Control and *AQP11* siRNAs (100 pmol/L, final concentration) were complexed with 5 µL of Lipofectamine^®^ 2000 reagent (Invitrogen) in serum-free Opti-MEM^®^ I (Invitrogen). After 20 min incubation at room temperature (RT), the mix was added to cells and incubated at 37 °C for 4 h. The transfection mixes were then completely removed and fresh adipocyte culture media were added. Knockdown effectiveness after 24 h was determined by real-time PCR.

### 2.6. Real-Time PCR

Transcript levels for AQP11 (*AQP11*), activating transcription factor 4 (*ATF4*), and DNA damage-inducible transcript 3 (*DDIT3*) were quantified by real-time PCR (7300 Real-Time PCR System; Applied Biosystems, Foster City, CA, USA). Primers and probes (Supplementary Table S1) were designed using the software Primer Express 2.0 (Applied Biosystems) and purchased from Genosys (Sigma). Primers or TaqMan^®^ probes encompassing fragments of the areas from the extremes of two exons were designed to ensure the detection of the corresponding transcript avoiding genomic DNA amplification. The cDNA was amplified at the following conditions: 95 °C for 10 min, followed by 45 cycles of 15 s at 95 °C and 1 min at 59 °C, using the TaqMan^®^ Universal PCR Master Mix (Applied Biosystems). The primer and probe concentrations were 300 and 200 nmol/L, respectively. All results were normalized for the expression of *18S* rRNA (Applied Biosystems), and relative quantification was calculated using the 2^−∆∆Ct^ formula [27]. Relative mRNA expression was expressed as fold expression over the calibrator sample. All samples were run in triplicate and the average values were calculated.

### 2.7. Western-Blot

Samples (30 μg) were run out in 10% SDS-PAGE, transferred to nitrocellulose membranes (Bio-Rad Laboratories, Inc., Hercules, CA, USA) and blocked in Tris-buffered saline (10 mmol/L Tris-HCl, 150 mmol/L NaCl, pH 8.00) with 0.05% Tween 20 (TBS-T) containing 5% non-fat dry milk for 1 h at RT. Blots were then incubated overnight at 4 °C with rabbit polyclonal anti-human AQP11 (HPA042879, Sigma) or murine monoclonal anti-β-actin (Sigma) antibodies (diluted 1:1000 and 1:5000, respectively, in blocking solution). The antigen-antibody complexes were visualized using horseradish peroxidase-conjugated anti-rabbit or anti-mouse IgG antibodies (diluted 1:5000 in blocking solution) and the enhanced chemiluminescence ECL Plus detection system (Amersham Biosciences, Buckinghamshire, UK). The band intensities were determined by densitometric analysis with the Gel Doc^TM^ system and Quantity One 4.5.0 software (Bio-Rad) and normalized with β-actin density values.

### 2.8. Immunohistochemistry of AQP11

The indirect immunoperoxidase method was used to detect AQP11 in histological sections of omental and subcutaneous fat [24]. Sections of formalin-fixed paraffin-embedded adipose tissue (6 µm) were dewaxed in xylene, rehydrated in decreasing concentrations of ethanol and treated with 3% H_2_O_2_ (Sigma) in absolute methanol for 10 min at RT to quench endogenous peroxidase activity. Slides were blocked during 30 min with 1% murine serum (Sigma) diluted in Tris-buffer saline (TBS) (50 mmol/L Tris, 0.5 mol/L NaCl; pH 7.36) to prevent nonspecific adsorption. Sections were incubated overnight at 4 °C with rabbit polyclonal anti-human AQP11 (HPA042879, Sigma) antibody diluted 1:1000 in TBS. After washing three times (5 min each) with TBS, slides were incubated with DAKO Real^TM^ EnVision^TM^ anti-rabbit/mouse (K5007; Dako, Golstrup, Denmark) for 1 h at RT. The peroxidase reaction was visualized using a 0.5 mg/mL diaminobenzidine (DAB)/0.03% H_2_O_2_ solution diluted in 50 mmol/L Tris-HCl, pH 7.36, and Harris hematoxylin solution (Sigma) as counterstaining. Negative control slides without primary antibody were included to assess nonspecific staining.

### 2.9. Confocal Immunofluorescence Microscopy of AQP11

Differentiated adipocytes grown on glass coverslips were fixed with 4% formaldehyde for 15 min at RT, incubated with PBS containing 0.3% saponin and 1% BSA for 1 h at RT, and exposed to rabbit polyclonal anti-human AQP11 antibody (HPA042879, Sigma) diluted 1:100 in PBS containing 0.3% saponin and 0.5% BSA overnight at 4 °C. Thereafter, cells were washed with PBS and incubated with Alexa Fluor^®^ 488-conjugated donkey anti-rabbit IgG (Invitrogen) diluted 1:500 for 2 h at RT. After washing, coverslips were mounted on microscope slides and examined under a TCS-SP2-AOBS confocal laser scanning microscope (Leica Corp., Heidelberg, Germany). After acquisition, images underwent a deconvolution process with the Huygens Essential 2.4.4 software (Scientific Volume Imaging, Hilversum, The Netherlands).

### 2.10. Statistical Analysis

Statistical analyses were performed using the SPSS 15.0 software. Data are expressed as the mean ± SEM. Statistical differences between mean values were analyzed using Student’s *t*-test, χ^2^ test, one-way ANOVA followed by Scheffe’s or Dunnett’s post hoc tests, or two-way ANOVA, where appropriate. Pearson correlation coefficients (*r*) were used to analyze the association between variables. A *p*-value <0.05 was considered statistically significant.

## 3. Results

### 3.1. Obesity and Obesity-Associated Type 2 Diabetes Upregulated AQP11 Expression in Human Visceral Fat

The clinical characteristics of our cohort are summarized in Table 1. Fifteen patients (22.4%) were on antihypertensive treatment, four subjects (5.9%) were on oral antidiabetic drugs, and six individuals (9.0%) were on lipid-lowering medications. No differences in age between groups were observed (*p* = 0.140). As expected, markers of adiposity (BMI, BF, or leptin) and lipolysis [circulating free fatty acids (FFA) and glycerol] were increased (*p* < 0.01) in both groups of patients with obesity compared to those of the lean individuals. Analogously, obese patients with T2D exhibited higher (*p* < 0.01) glycemia, insulinemia, HOMA, and Adipo-IR indices, as well as a lower QUICKI index (*p* < 0.001). Obesity was also associated with an abnormal lipid profile, evidenced by increased reduced levels of HDL-cholesterol (*p* < 0.05). Moreover, patients with obesity exhibited increased circulating levels of markers of systemic inflammation, including C-reactive protein (CRP), fibrinogen, von Willebrand factor, or TNF-α (all *p* < 0.05), regardless of insulin resistance.

**Figure 1:**
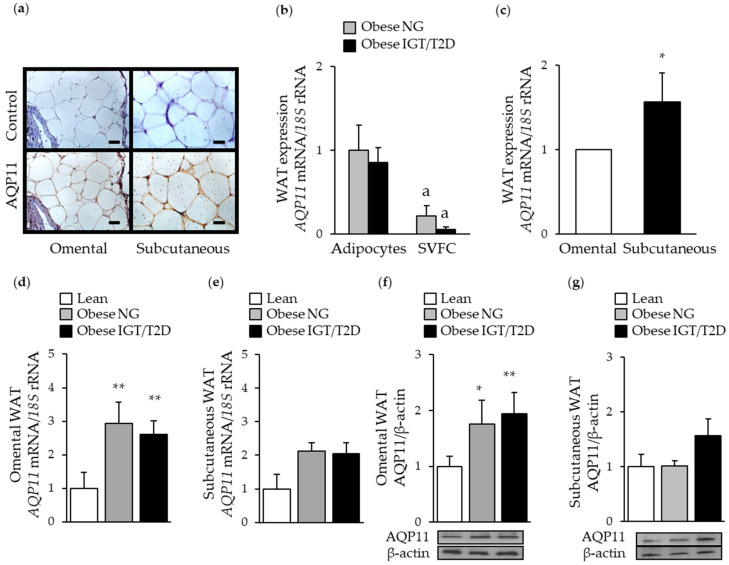
Impact of obesity and obesity-associated T2D on AQP11 expression in paired omental and subcutaneous adipose tissue samples. (**a**) Immunohistochemical detection of AQP11 in omental (left panels) and subcutaneous (right panels) fat depots obtained from patients with obesity (magnification, ×200; scale bar = 50 µm). Comparison of mRNA levels of *AQP11* in freshly isolated adipocytes and SVFC from omental WAT from patients with obesity classified according to their degree of insulin resistance (**b**) as well as in human omental and subcutaneous white adipose tissue (WAT) (**c**). Bar graphs show the expression of AQP11 mRNA and protein in omental (**d** and **f**) and subcutaneous (**e** and **g**) WAT obtained from lean individuals, obese patients with normoglycemia (NG), impaired glucose tolerance (IGT), or type 2 diabetes (T2D). Representative blots are shown at the bottom of the histograms. * *p* < 0.05, ** *p* < 0.01 vs. mRNA expression in omental WAT or in lean subjects. ^a^ *p* < 0.05 vs. mRNA expression in the adipocyte fraction.

First, we assessed the expression of AQP11 in paired omental and subcutaneous adipose tissue samples by real-time PCR, Western-blot, and immunohistochemistry. The tissue distribution of AQP11 in biopsies of omental and subcutaneous adipose tissue showed predominant brown immunostaining in fully mature adipocytes (Figure 1a). Accordingly, adipose tissue fraction analysis also revealed an increase (*p* <0.05) in *AQP11* mRNA levels in adipocytes in comparison to SVFC, independently of the insulin resistance degree (Figure 1b). Lack of changes (*p* = 0.329) in *AQP11* mRNA expression after insulin treatment confirmed that this hormone is not a major regulator of AQP11 in human adipocytes (Figure S1). We next examined potential fat depot-differences in *AQP11* mRNA expression. *AQP11* was expressed to a greater (*p* < 0.05) extent in the subcutaneous adipose tissue (Figure 1c). However, obesity and obesity-associated T2D were associated with increased (*p* < 0.05) AQP11 mRNA and protein levels in omental adipose tissue (Figure 1d,f), without changes in subcutaneous fat (Figure 1e,g). AQP11 protein expression levels in visceral fat were positively correlated with markers of adiposity, such as BMI (*r* = 0.48, *p* = 0.004), body fat (*r* = 0.49, *p* = 0.004), and leptin (*r* = 0.42, *p* = 0.016), and with the acute-phase reactant CRP (*r* = 0.37, *p* = 0.041), suggesting an involvement of this superaquaporin in obesity-associated inflammation.

### 3.2. AQP11 is Increased during Adipocyte Differentiation and Lipolysis

**Figure 2:**
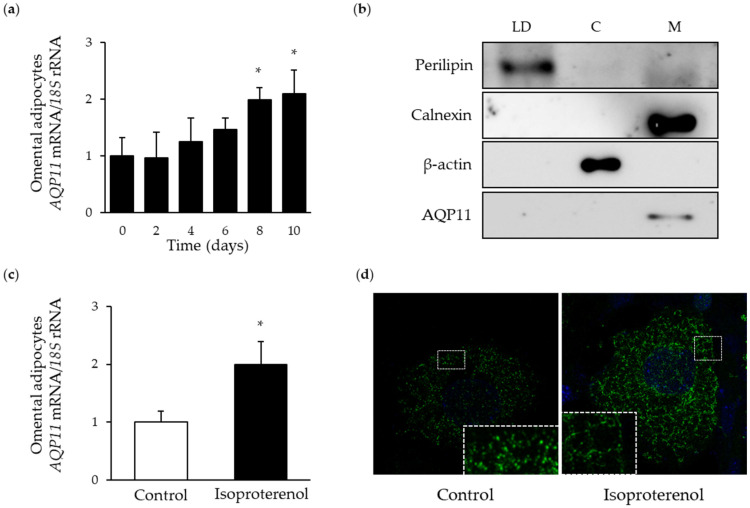
Characterization of AQP11 during adipocyte differentiation and lipolysis. (**a**) Time course of *AQP11* mRNA expression during adipocyte differentiation. (**b**) AQP11 protein expression in differentiated adipocyte fractions corresponding to lipid droplets (LD), cytosol (C) or membranes (M); perilipin, β-actin, and calnexin were used as markers for lipid droplets, cytoplasm, or endoplasmic reticulum membrane, respectively. AQP11 mRNA expression (**c**) and protein redistribution (**d**) after 24 h treatment with isoproterenol (10 µmol/L) in human differentiated adipocytes. Gene expression in SVFC in day 0 or control adipocytes were assumed to be 1. * *p* < 0.05 vs. mRNA expression in omental WAT; * *p* < 0.05 vs. mRNA expression in SVFC in day 0 or control adipocytes.

To gain further insight into the role of AQP11 in the onset of obesity, we evaluated the expression and subcellular location of this superaquaporin during adipocyte differentiation and lipolysis. In line with the above-mentioned results, AQP11 transcription was increased during late adipocyte differentiation (Figure 2a), confirming that mature adipocytes are the major source of AQP11 in human adipose tissue. Under basal conditions, AQP11-positive immunosignal was observed as puncta around and between lipid droplets (Figure 2d). Subcellular fractionation studies of differentiated adipocytes revealed that AQP11 mostly associated with ER-enriched cellular membranes, as indicated by the presence of the ER marker calnexin (Figure 2b) and not in fractions containing lipid droplets (immunolabeled for perilipin) or cytosol (immunolabeled for β-actin). Upon 24 h stimulation with isoproterenol, *AQP11* mRNA was increased (*p* < 0.05) (Figure 2c), and protein tended to appear more concentrated around the lipid droplets (Figure 2d).

### 3.3. AQP11 Participates in TGF-β1-Induced Endoplasmic Reticulum Stress

**Figure 3:**
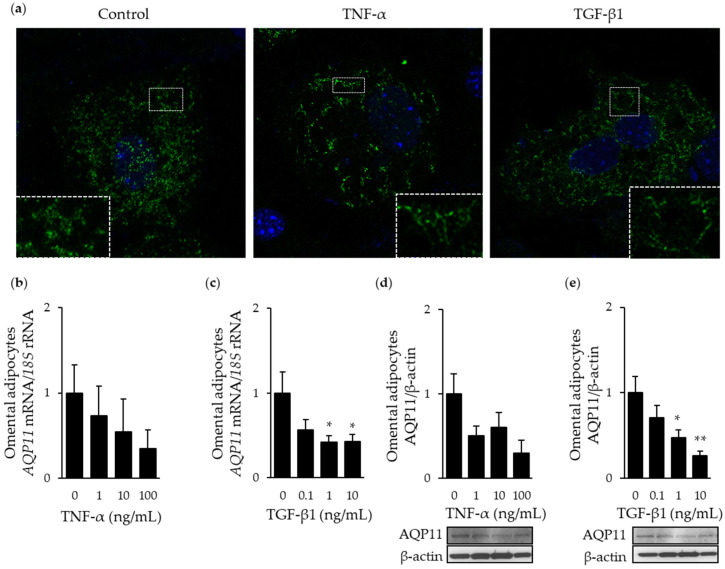
Proinflammatory factors TNF-α and TGF-β1 induce changes in AQP11 expression and subcellular distribution. (**a**) Immunocytochemical detection of AQP11 in differentiated adipocytes (day 10) under basal conditions (left panel) and after the stimulation for 24 h with TNF-α (10 ng/mL) (*middle panel*) or TGF-β1 (10 ng/mL) (right panel). Bar graphs show AQP11 mRNA (**b** and **c**) and protein (**d** and **e**) after 24 h treatment with different concentrations of TNF-α and TGF-β1 in differentiated omental adipocytes. Representative blots are shown at the bottom of the histograms. * *p* < 0.05, ** *p* < 0.01 vs. unstimulated cells.

AQP11 also acts as a peroxiporin for the efflux of cellular H_2_O_2_ and alleviation of ER stress [10]. Since TNF-α and TGF-β1 are major inductors of inflammation and ER stress in the adipose tissue, we next analyzed their impact on AQP11 expression and subcellular distribution in adipocytes. Confocal microscopy studies revealed lower immunostaining of AQP11 in adipocytes stimulated with TNF-α (10 ng/mL) or TGF-β1 (10 ng/mL) for 24 h (Figure 3a), with the distribution of this superaquaporin appearing more prominently surrounding the lipid droplets. Adipocytes treated with different concentrations of TNF-α exhibited lower mRNA (*p* = 0.074) and protein (*p* = 0.144) expression of AQP11, but differences fell out of statistical significance (Figure 3b,d). Increasing concentrations of TGF-β1 significantly diminished the transcript (*p* = 0.035) and protein (*p* = 0.004) levels of AQP11 in adipocytes (Figure 3c,d).

**Figure 4:**
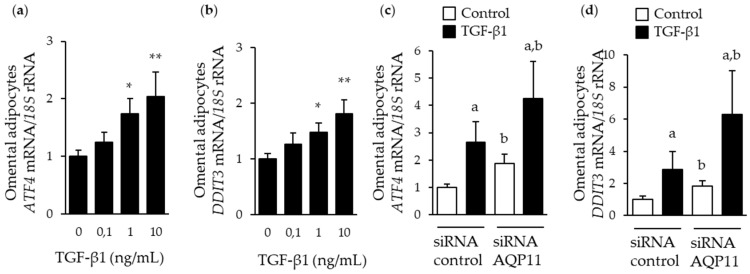
Impact of *AQP11* gene silencing on basal and TGF-β1-induced ER stress. Bar graphs show *ATF4* and *DDIT3* transcript levels in omental adipocytes after 24 h treatment with different concentrations of TGF-β1 (**a** and **b**) as well as in *AQP11*-silenced adipocytes after stimulation with TGF-β1 10 ng/mL for 24 h (**c** and **d**). * *p* < 0.05, ** *p* < 0.01 vs. unstimulated cells; ^a^ *p* < 0.05 effect of TGF-β1 treatment; ^b^ *p* < 0.05 effect of *AQP11* gene silencing.

Since TGF-β1 appears to be a major regulator of AQP11 in adipocytes, we focused the next experiments on this proinflammatory factor. Given their relevance in the context of obesity, we next analyzed the expression of the genes encoding ER stress markers ATF4 and C/EBP homologous protein (CHOP) (*ATF4* and *DDIT3*) [20,28]. As expected, TGF-β1 induced an increased (*p* < 0.05) the expression of these ER stress markers at the concentrations of 1 and 10 ng/mL (Figure 4a,b). To evaluate whether AQP11 is necessary for TGF-β1-induced ER stress, we downregulated the constitutive expression levels of *AQP11* in human omental adipocytes using a pool of siRNAs targeting *AQP11* mRNA, getting an average knockdown of 92% (Figure S2). *AQP11* gene silencing upregulated (*p* < 0.05) in *ATF4* and *DDIT3* mRNA levels under basal conditions, and further aggravated (*p* < 0.05) TGF-β1-induced activation of both ER stress markers (Figure 4c,d).

## 4. Discussion

**Figure 5:**
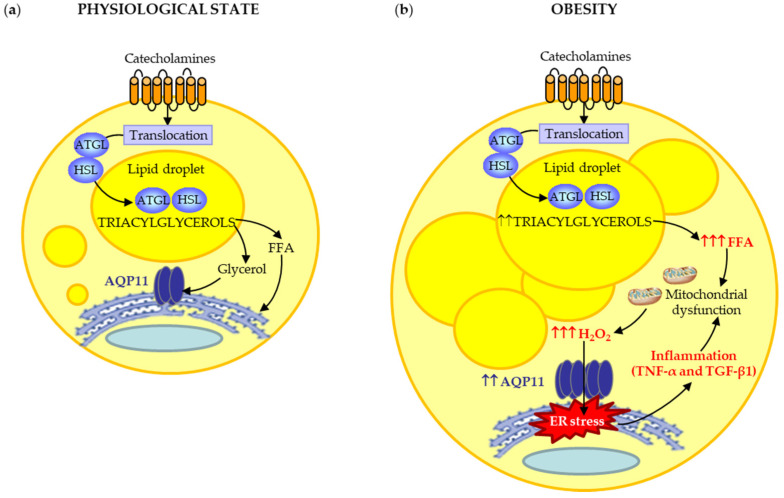
(**a**) Under physiological conditions, AQP11 contributes to glycerol mobilization for triacylglycerol synthesis in nascent lipid droplets in the ER. (**b**) In obesity, the peroxiporin activity of AQP11 appears to contribute to the alleviation of ER stress induced by the increased ROS production due to mitochondrial dysfunction under lipotoxic and inflammatory conditions. Although proinflammatory factors, such as TNF-α, TGF-β1, or LPS downregulate AQP11, this superaquaporin is upregulated in obesity, suggesting that other factors are involved in AQP11 regulation in human adipocytes. ATGL, adipocyte triglyceride lipase; HSL, hormone-sensitive lipase.

AQP11 is expressed mainly in mature adipocytes of omental and subcutaneous adipose tissue. Our results showed an increase in AQP11 expression during adipocyte differentiation, a process accompanied by lipid droplet (LD) biogenesis. In adipocytes, nascent LDs arise from the bilayer membrane of the ER [29]. A previous study revealed that AQP11 partially colocalizes with perilipin-1 [9], a constitutive LD-coating protein that seems to move between LDs and ER [30,31]. Accordingly, our data confirmed that AQP11 is located in the ER membrane at the vicinity of LDs, suggesting its potential contribution to glycerol uptake for triacylglycerol synthesis within the ER-emerging LDs (Figure 5a). The functionality of AQP11 as a glycerol channel was evidenced by its responsiveness to isoproterenol-induced lipolysis, a catabolic process leading to triacylglycerol breakdown into glycerol and FFA [32]. The β-adrenergic agonist isoproterenol stimulates glycerol efflux from adipocytes triggering the translocation of aquaglyceroporins from the cytosolic fraction (AQP3) or lipid droplets (AQP7 and AQP10) to the plasma membrane [24,33,34,35]. Notably, our confocal microscopy studies revealed that isoproterenol reinforced the presence of AQP11 around LDs, instead of promoting its trafficking towards the plasma membrane, like aquaglyceroporins do. In this sense, lipolysis induces LD shrinkage and fragmentation into microLDs during triacylglycerol breakdown [36]. Thus, the marked presence of AQP11 around LDs after isoproterenol-induced lipolysis might reflect an efficient glycerol mobilization during microLD formation in the ER under physiological conditions (Figure 5).

In obesity, catecholamine-induced lipolysis is markedly increased in visceral fat due to increased activity of lipolytic β-adrenergic receptors and decreased activity of anti-lipolytic α_2_-adrenoceptors [32]. Accordingly, circulating levels of glycerol and FFA were observed in both obese groups of our cohort. Interestingly, the sustained elevation of FFA in the obese state has the potential to increase reactive oxygen species (ROS) production and ER stress in adipocytes [21]. In this regard, ROS oxidizes nascent proteins and increases misfolded and unfolded proteins in the ER. Although all organelles and cell compartments produce ROS, mitochondrial generation of H_2_O_2_ is generally considered to be the major source of oxidants. ROS, and particularly H_2_O_2_, overwhelm the ER structure and chaperone activity, initiating a vicious circle of stress, mitochondrial dysfunction, and ER stress worsening [20,21,22]. The ER and mitochondria join at multiple contact sites to form specific domains, termed mitochondria-ER associated membranes (MAMs), which prompt danger signal sensing and inflammatory responses. Interestingly, AQP11 accumulates partly in MAMs in HeLa cells [10]. Thus, it seems plausible that the herein observed upregulation of AQP11 mRNA and protein levels in visceral fat might constitute a compensatory mechanism to protect adipocytes against ER stress induced by lipotoxicity in obesity (Figure 5). Further investigations analyzing the role of AQP11 in ER-mitochondria contact sites (MAMs) to sense the level of danger and to coordinate the appropriate inflammatory response in visceral adipocytes are warranted.

Growing evidence supports that obesity-associated inflammation and ER stress take also place in the subcutaneous fat depot [37]. In the context of obesity-associated insulin resistance, subcutaneous adipose tissue can be as deleterious as visceral fat, exhibiting increased markers of inflammation, oxidative, and ER stress [25]. Accordingly, our data showed that transcript and protein levels of AQP11 were higher in the subcutaneous adipose tissue of obese patients with type 2 diabetes than those found in individuals with normoglycemia. However, no significant differences were found, which might be ascribed to the short diabetes history (<2–3 years or even de novo diagnosis) of our cohort. Future studies in patients with obesity and a longer type 2 diabetes history are warranted to address this point.

Chronic UPR activation in adipocytes is directly linked to the activation of inflammatory signaling pathways that regulate the transcription of proinflammatory cytokines, including TNF-α and TGF-β1 [38]. Moreover, the simultaneous activation of inflammation and mitochondrial dysfunction trigger ROS production, perpetuating ER stress in the context of obesity [38,39]. In the present study, inflammatory factors TNF-α, and especially TGF-β1, decreased AQP11 mRNA and protein in human visceral adipocytes. TNF-α has been also reported to decrease the protein expression of another aquaglyceroporin, AQP9, in a hepatocyte model of fatty liver disease [40]. IKK/NF-κB participates in the proinflammatory signal transduction induced by ER stress during adipocyte dysfunction [41]. In line with this observation, Chiadak and colleagues [42] found that the exogenous inflammatory factor LPS downregulates *Aqp11* mRNA levels in 3T3-L1 adipocytes through TLR4-induced activation of JNK and NF-κB pathways. Thus, alterations in the IKK/NF-κB signal might also be involved in ER stress during *AQP11* silencing. Importantly, *AQP11* gene silencing induced an increased basal and TGF-β1 expression of ER stress markers, ATF4 and CHOP, supporting the crucial role of AQP11 in ER homeostasis in human visceral adipocytes. Together, it seems plausible that, in obesity, the overexpression of AQP11 also alleviates inflammation-induced ER stress in visceral adipocytes due to its peroxiporin activity (Figure 5b).

## 5. Conclusions

In summary, our data sheds light into novel functions of AQP11 due to its ability to transport glycerol and H_2_O_2_ through the ER membrane in human adipose tissue (Figure 5). Under physiological conditions, AQP11 is an ER-resident protein that allows glycerol mobilization for the synthesis of triacylglycerols in nascent lipid droplets in the basal state or after β-adrenergic lipolytic stimulation. However, in the obese state, its overexpression in the visceral adipose tissue appears to be a compensatory mechanism to alleviate ER stress due to its ability to transport H_2_O_2_. In this regard, the downregulation of AQP11 in visceral adipocytes aggravates TGF-β1-induced ER stress, as evidenced by the increased ER markers ATF4 and CHOP. Targeting AQP11 to reduce ER stress might constitute a potential therapeutic target for the treatment of obesity.

## Figures and Tables

**Table 1:** Clinical characteristics of the subjects of the study.

	Lean	Obese NG	Obese IGT/T2D	*p*
*n*	14	24	29	-
Sex (male/female)	6/8	10/14	13/16	0.973
Age (years)	48 ± 3	41 ± 3	45 ± 2	0.140
BMI (kg/m^2^)	23.1 ± 0.8	47.2 ± 1.5 ^a^	48.6 ± 1.6 ^a^	**<0.0001**
Body fat (%)	24.8 ± 2.8	51.7 ± 1.4 ^a^	52.1 ± 1.3 ^a^	**<0.0001**
Glucose (mg/dL)	85 ± 3	91 ± 2	120 ± 7 ^a,b^	**0.001**
Glucose 2-h OGTT (mg/dL)	-	119 ± 6	194 ± 14 ^b^	**<0.0001**
Insulin (µU/mL)	7.8 ± 1.4	21.1 ± 2.8 ^a^	23.9 ± 4.9 ^a^	**0.004**
Insulin 2-h OGTT (µU/mL)	-	93.2 ± 13.6	90.6 ± 7.3	0.862
HOMA	1.7 ± 0.3	4.8 ± 0.7 ^a^	7.8 ± 2.0 ^a^	**0.007**
QUICKI	0.36 ± 0.01	0.31 ± 0.01 ^a^	0.31 ± 0.01 ^a^	**0.014**
FFA (mmol/L)	13.3 ± 1.6	17.0 ± 1.3	18.6 ± 1.9 ^a^	**0.040**
Glycerol (mg/dL)	22.5 ± 3.5	34.1 ± 3.4	44.4 ± 5.0 ^a^	**0.008**
Adipo-IR index	21.1 ± 3.1	83.6 ± 11.6	108.6 ± 19.8 ^a^	**0.003**
Triacylglycerol (mg/dL)	68 ± 9	135 ± 19 ^a^	165 ± 33 ^a^	**0.011**
Total cholesterol (mg/dL)	191 ± 8	196 ± 8	200 ± 6	0.800
LDL-cholesterol (mg/dL)	117 ± 8	119 ± 8	130 ± 6	0.448
HDL-cholesterol (mg/dL)	59 ± 2	49 ± 5 ^a^	44 ± 2 ^a^	**0.013**
CRP (mg/L)	2.3 ± 0.6	8.8 ± 1.4 ^a^	11.5 ± 2.8 ^a^	**0.001**
Uric acid (mg/dL)	4.2 ± 0.4	9.2 ± 2.8 ^a^	6.5 ± 0.2 ^a^	**0.007**
Leptin (ng/mL)	7.2 ± 1.4	46.9 ± 5.8 ^a^	53.5 ± 6.5 ^a^	**0.004**
TNF-α (ng/mL)	0.87 ± 0.15	1.89 ± 0.12 ^a^	2.02 ± 0.41 ^a^	**0.003**
Fibrinogen (mg/dL)	251 ± 42	358 ± 16 ^a^	372 ± 15 ^a^	**0.003**
von Willebrand factor (%)	87 ± 11	126 ± 9 ^a^	154 ± 14 ^a^	**0.025**
Antihypertensive therapy, *n* (%)	0 (0%)	7 (29%)	8 (28%)	0.092
Antidiabetic therapy, *n* (%)	0 (0%)	0 (0%)	4 (14%)	**0.041**
Lipid-lowering therapy, *n* (%)	0 (0%)	4 (17%)	2 (22%)	0.384

NG, normoglycemia; IGT, impaired glucose tolerance; T2D, type 2 diabetes; BMI, body mass index; OGTT, oral glucose tolerance test; HOMA, homeostasis model assessment; QUICKI, quantitative insulin sensitivity check index; FFA, free fatty acids; Adipo-IR, adipocyte insulin resistance index; CRP, high-sensitivity C-reactive protein; TNF-α, tumor necrosis factor α. Differences between groups were analyzed by one-way ANOVA followed by a Scheffe’s test or Student’s *t*-test or χ^2^ test, where appropriate. Bold values denote statistically significant *p* values. ^a^ *p* < 0.05 vs. normal-weight individuals; ^b^ *p* < 0.05 vs. obese NG patients.

## Supplementary Materials

The following are available online at https://www.mdpi.com/2073-4409/9/6/1403/s1, Figure S1: Effect of insulin treatment on transcript levels of *AQP11* in human differentiated adipocytes. Figure S2: Efficiency of *AQP11* gene knockdown using MISSION^®^ siRNA treatment. Table S1: Sequences of primers and TaqMan^®^ probes.
