# Supplementary material for: Aquaporin-11 Contributes to TGF-β1-induced Endoplasmic Reticulum Stress in Human Visceral Adipocytes: Role in Obesity-Associated Inflammation
Source: Cells. 2020 Jun 4;9(6):1403. doi: 10.3390/cells9061403 (PMC7349025; doi:10.3390/cells9061403)
Supplement: Supplementary file 1 [file cells-09-01403-s001.pdf]

Table S1. Sequences of primers and TaqMan® probes.

Gene (GenBank accesion no.)	Oligonucleotide sequence (5′-3′)	Nucleotides
AQP11		
(NM_173039.2)		
Forward	TTCCAGGAAGTCCGAACCAA	908-927
Reverse	GCTTTGGCACTTTCGCTACATT	1009-1031
Probe	FAM-TTTGGTCTATGCAGGAGGAAGTCTAAC-TAMRA	964-990
ATF4		
(NM_001675.4)		
Forward	AGGTGGCCAAGCACTTCAAA	1003-1022
Reverse	CAACAACAGCAAGGAGGATGC	1099-1120
Probe	FAM-TCATGGGTTCTCCAGCGACAAGGCT-TAMRA	1025-1049
DDT3		
(NM_001195053.1)		
Forward	CCTGGAAATGAAGAGGAAGAATCA	461-484
Reverse	TCACAAGCACCTCCCAGAGC	554-574
Probe	FAM-CTTGACCCTGCTTCTCTGGCTTGGCT-TAMRA	500-525

AQP11, aquaporin-11; *ATF4,* activating transcription factor 4; *DDT3,* DNA-damage-inducible transcript 3.

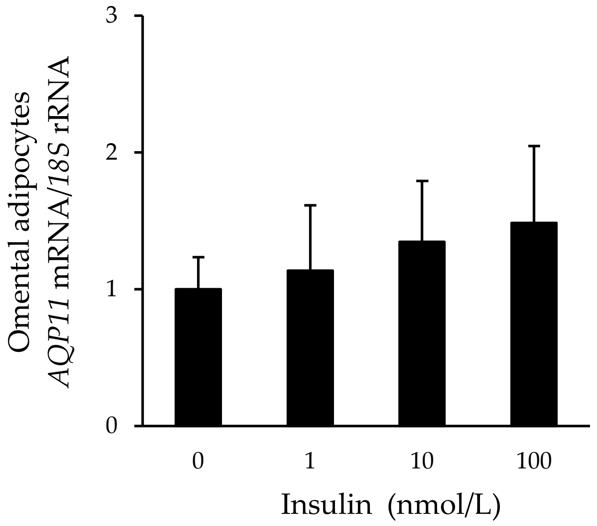

Fig. S1. Effect of insulin treatment on transcript levels of *AQP11* in human differentiated adipocytes. Gene expression in unstimulated cells was assumed to be 1.

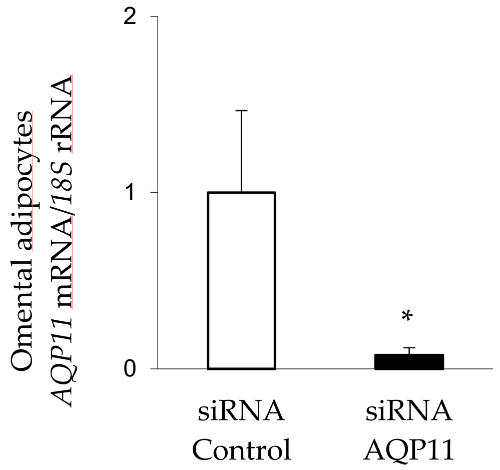

Fig. S2. Efficiency of *AQP11* gene knockdown using MISSION® siRNA treatment. *AQP11* mRNA levels in human omental differentiated adipocytes after knockdown of *AQP11* expression with a pool of siRNA for 24 h. Differences between groups were analyzed by Student's *t* test. *P<0.05 *vs* control siRNA cells.
